# Mapping Interactions Within EU Food and Feed Risk Analysis: A Comparative Study of Risk Assessors and Risk Managers

**DOI:** 10.1111/risa.70376

**Published:** 2026-10-05

**Authors:** Axel Menning, Leonie Dendler, Joao Borges, Anneluise Mader, Mats Gunnar Andersson

**Affiliations:** ^1^ German Federal Institute for Risk Assessment (BfR) Berlin Germany; ^2^ Wageningen University Wageningen the Netherlands; ^3^ National Veterinary Institute (SVA) Uppsala Sweden

**Keywords:** empirical research, enterprise risk management, multivariate analysis of variance, multivariate analysis, perception, regression analysis, risk analysis, risk assessment, risk management tools, risk management

## Abstract

Considering prominent calls for various forms of cooperation, coordination, and collaboration within risk analysis processes, this empirical research study aims to delineate current forms of interaction between risk assessors and risk managers. Utilizing an online survey, it analyses responses from 95 professionals working in the field of food and feed risk analysis across at least 23 EU countries and determines interaction scores based on the degree of cooperation, coordination, and collaboration. The scores show that current interaction dominantly aligns with cooperation across multiple dimensions. However, multivariate analysis of variance (MANOVA) reveals significant perceptual differences between risk assessors and managers regarding their mutual interaction. In particular, risk assessors perceive their interaction with risk management more cooperative and risk managers perceive their interaction with risk assessment more collaborative. A notable difference was also observed in the interaction scores for routine risk analysis versus rapid risk analysis. In addition, clustering analysis and regression analysis was conducted to examine the relation and impact of different interaction dimensions on perceived quality of risk analysis. While the results do not support uniform calls for more collaboration, they show positive effects of open and frequent communication on the perceived quality of risk analysis. These results suggest working towards more individualized and fit‐for‐purpose interactions as well as to extend practitioner‐oriented dialogue on desired modes of interactions, to enhance mutual understanding in their collective risk analysis.

## 1 Introduction

The formal definition of risk communication, as established by frameworks such as the Joint FAO/WHO Codex Alimentarius Commission (2023), encompasses an interactive exchange of information and opinions among a wide array of stakeholders, including risk assessors, risk managers, consumers, industry, and the academic community. Fulfilling the comprehensive vision of this framework requires robust internal integration across all administrative channels. Accordingly, scholars and policymakers continue to emphasize the critical need for examining the operational interaction among core participants in the risk analysis cycle—particularly between risk assessors and risk managers—to clarify how the systems map onto paradigms of cooperation, coordination, and collaboration. In a recent summary of the “One Society” track of the “ONE—Health, Environment & Society—Conference” organized in 2022 by major European risk agencies, Frewer et al. 2023, 2) highlight the need “to realize the EU's collaborative food safety knowledge ecosystem.” This resonates with calls from many authors from the wider public administration literature to move away from top‐down, siloed decision making toward more horizontal, networked approaches (Costumato 2021; Emerson et al. 2011; Osei‐Kojo et al. 2020).

The analysis of potential health risks associated with the food and feed chain is crucial to ensure the provision of safe food to consumers. While the detailed organization of food and feed risk analysis differs across Europe (Abels and Kobusch 2015; Vos and Wendler 2006), it is defined by regulation as a process consisting of three interconnected components: risk assessment (RA), risk management (RM), and risk communication (EC General Food Law Regulation No. 178/2002, Art. 3[10]). These components involve distinct tasks and require different skill sets, indicating that multiple actors and organizations are involved in the risk analysis process. The distinction and distribution of tasks and competencies are central aspects manifested in the General Food Law Regulation to ensure the autonomy of the organizations involved. However, while clear roles and activities are outlined, there are potential limitations to this demarcation in practice.

To properly contextualize this empirical research study, it is important to note that our research is strictly situated within the European Union (EU) regulatory framework, which operates under different administrative and legal structures compared to other jurisdictions, such as the United States. Under the EU General Food Law (Regulation (EC) No 178/2002), risk analysis is designed with a functional separation between RA and RM. Within this specific European architecture, “risk assessors” are the scientific experts and independent agencies (e.g., the European Food Safety Authority [EFSA] and national equivalents) tasked with scientific evaluation. Conversely, “risk managers” refer explicitly to the regulatory, executive, and standard‑setting authorities (e.g., the European Commission and national competent authorities) who weigh policy alternatives and enact regulations. In the context of this paper, the term “risk manager” does not encompass industry actors, manufacturers, or food producers, but rather focuses strictly on these governmental and regulatory bodies. We acknowledge that RM in its broadest sense is heavily reliant on the producers, manufacturers, and retailers who execute these controls on the ground. Minimizing this dimension carries the structural danger of developing policies unaligned with real‑world technical and operational limitations. Furthermore, public risk managers are legally and structurally mandated to solicit input and baseline compliance data directly from regulated industry bodies during policy formulation. Moreover, in an interconnected global agrifood trade system, internal EU RM decisions do not operate in a vacuum. Under World Trade Organization (WTO) mechanisms—specifically the Agreements on the Application of Sanitary and Phytosanitary Measures (SPS) and Technical Barriers to Trade (TBT)—regulations enacted by EU risk managers can be formally scrutinized or contested by non‐EU trading partners to prevent unjustified non‐tariff trade barriers or discriminatory domestic preferences. Demonstrating an objective, transparent, and methodologically sound scientific assessment underpinning managerial decisions is paramount to upholding these regulatory measures against international legal challenges. While our investigation remains explicitly bounded to the internal public administration interface between regulatory assessors and managers, we emphasize that this public interface operates within a wider network requiring robust industry data flow and alignment with multilateral trade disciplines. In this paper, the term “interaction” is used as an umbrella term representing any form of contact, communication, or information exchange between RA and RM, which can take place within various organizational designs, including strict functional separation.

Traditionally, the risk analysis process has been conceptualized through a highly structured workflow—often initiating with a request from RM (RM), proceeding to RA for scientific evaluation, and returning to RM for final decision‐making. While this foundational model has always accommodated necessary communication between assessors and managers, the increasing complexity of modern food and feed risks has prompted discussions on how to optimize this workflow. Building on these established communication channels, scholars, and policymakers are increasingly exploring whether more iterative and continuous forms of interaction might further enhance the process, without compromising the distinct responsibilities of each group (IRGC 2017; Thompson and Bloom 2000; Vareman and Persson 2010). Indeed, calls for improved RA–RM coordination have persisted for decades (Robinson and Levy 2011, 1334; Greenberg et al. 2015). Especially when risks are complex, ambiguous, or systemic, a more interactive approach is frequently recommended (IRGC 2017; Thompson and Bloom 2000; Vareman and Persson 2010; Robinson and Levy 2011). Importantly, advocating for robust interaction does not imply that interaction and functional separation are structurally incompatible. As explicitly established in foundational frameworks like the Codex Alimentarius, the maintenance of a clear functional separation is fully designed to coexist with—and indeed relies upon—an active, multi‐directional exchange of information and opinions.

True iterative collaboration introduces distinct temporal and procedural challenges. For interaction to be genuinely collaborative, scientific RAs must frequently be revisited or updated to gauge the precise impact of various RM options under consideration. This iterative feedback loop can dramatically extend the timeframe needed to finalize a regulatory approach. Furthermore, focusing solely on the immediate mandate overlooks the broader regulatory lifecycle; for major requirements, the process typically originates five to ten years prior to the formal RA phase. This lengthy preliminary stage is dedicated to identifying available scientific data, funding targeted research, and acquiring technical inputs from the relevant food and feed industries to build the scientific foundation required for the subsequent assessment.

However, such calls raise tensions with the prevalent understanding of risk analysis, which emphasizes a clear separation between RA and RM. This separation was purposefully designed to protect scientific RA from political bias and to maintain public trust. This foundational architecture has historically served as a robust and highly successful institutional safeguard for ensuring scientific objectivity. The exploration of more continuous interactions is therefore not an indication of a poorly designed system, but rather an ongoing discussion on how to optimize workflows to address increasingly complex hazards while maintaining the structural integrity and clear boundaries of the existing functional separation.

The US National Research Council's “Red Book” (1983, 7) famously recommended “that regulatory agencies take steps to establish and maintain a clear conceptual distinction between assessment of risks and consideration of risk management alternatives.” While the Red Book acknowledged that RA and RM must “interact, and communication in both directions is desirable,” its call for a clear boundary quickly became institutionalized. Today, the debate over this boundary persists globally. In the United States, agencies like the FDA and EPA have historically faced criticism for maintaining a strict, internal “wall” between their scientific assessors and regulatory decision‐makers—a division often attacked as an “artificial dichotomy” that paralyzes risk‐management action (NRC 2009; Robinson and Levy 2011). Similarly, while China's Food Safety Law established a strict functional separation between RA and RM, Wu et al. (2018) demonstrate that without formal, iterative interaction during the “framing” and “evaluation” steps, regulatory decisions are severely delayed. These global parallels suggest that the EU's ongoing challenges with RA–RM integration are neither unique nor inevitable.

Since the publication of the “Red Book,” the literature presents two primary structural perspectives regarding the RA–RM interface. On the one hand several works have advocated for a more interactive approach (Millstone 2009; Bier 2020; North 2020). Webler and Tuler (2021, 508) describe how the separation of science and politics has been increasingly broken down in the United States and Europe, particularly for risks with high complexities, ambiguities, and uncertainties around economic and wider social consequences. On the other hand, especially amongst practitioners, risk analysis often remains conceptualized as two discrete processes—scientific RA on one side, and a value‐laden political RM on the other (Hardy et al. 2020; Jasanoff 1993; Hoffmann 2011; Demortain 2023). Anderson et al. (2020, 2291) recently called in this journal for maintaining a strict separation of science and judgment “as originally called for in the Red Book,” underscoring the tension between different ideals and logics.

In the EU food‐and‐feed sector, this tension has historical roots. The BSE crisis drove policymakers to insulate scientific RA from political and economic influences in order to restore public trust (Ely et al. 2009; Holm and Halkier 2009; Schweizer 2021; Vareman and Persson 2010; Löfstedt et al. 2001; Vos and Wendler 2006). This led to the creation of the EFSA and widespread adoption of models with organizational separation between normal science‐based RA and political RM (Abels and Kobusch 2015). Yet scholars such as Hardy et al. (2020) describe this approach as “normalized” rather than ideal, suggesting room for evolution toward more collaborative structures, especially in case of emerging and novel risks.

In practice, calibrating interaction between risk assessors and managers involves a delicate balancing act: limited communication impedes the dynamic exchange needed for complex hazards, whereas unstructured interactions risk compromising scientific independence and public trust. In this study, we examine how EU food safety authorities navigate these competing pressures—upholding perceptions of scientific autonomy while fostering necessary RA–RM dialogue.

Yet, very little empirical research on the current state of interaction between risk assessors and risk managers exists. To explore these dynamics, we consider the following research questions:

1. How can the current state of interaction between risk assessors and risk managers be characterized?
  - Does the perception of the interaction vary between risk managers and risk assessors?
  - Does the category of risk analysis (rapid vs. routine RA) impact the perceptions of the interaction?
2. Can the risk analysis process be improved by specific forms of interaction?
  - Are different forms of interaction correlated with perceived quality of risk analysis?
  - Which form of interaction positively affects the perceived quality of risk analysis?
  - Which underlying interactional factors have an impact on the perceived quality of risk analysis?

We begin by reviewing existing literature to identify relevant frameworks that can serve as a foundation for analyzing and quantifying the interaction between RA and RM. We then present our survey involving 95 EU risk assessors and risk managers working in the field of food and feed safety to shed light on current interaction practices within the risk analysis process. We compare interaction forms and explore potential differences and effects in relation to risk analysis performance. We conclude with a reflection on barriers and facilitators for closer interaction and lessons learned for EU food and feed risk analysis. We also discuss the relevance of our findings for the wider risk analysis community.

## 2 Literature Review

Looking at the field of food and feed risk analysis, we find various calls for closer cooperation, coordination and/or collaboration within the risk analysis process as well as an emphasis on separating especially those involved in RA and those involved in RM: On the one hand the General Food Law, for example, defines risk communication as an “interactive exchange of information and opinions throughout the risk analysis process” (regulation (EC) No 178/2002). The Authority, Commission and Member States “shall cooperate to promote the effective coherence between risk assessment, risk management and risk communication functions” and “shall act in close collaboration […] to promote the necessary coherence in the risk communication process” (regulation (EC) No 178/2002). On the other hand, the same regulation postulates a clear distinction and distribution of risk analysis tasks and competences. This is to ensure the autonomy of participating organizations, where especially RM is supposed to be “distinct from risk assessment” with the latter being “based on the available scientific evidence and undertaken in an independent, objective, and transparent manner”. The more recent regulation on the transparency and sustainability of the EU RA in the food chain (2019/1381) continues this. It re‐emphasizes the need to guarantee the “independence” of risk assessors and risk managers while also calling to ʻʻestablish appropriate mechanisms of coordination and cooperation between the risk assessors and risk managers at Union and national level involved in the risk analysis process, in particular where several Union agencies provide scientific outputs on the same or on related subject matters, to ensure coherent risk communication and an open dialogue amongst all interested parties (European Food Safety Authority et al., 2021).

Improving interaction between organizational actors has been a longstanding topic in public administration and management (Keast et al. 2007; Lodge 2009; Onyango 2019). Yet, describing interpersonal interaction in organizational settings remains elusive. Terms used to characterize interaction often suffer from ambiguity. They are used interchangeably with little consideration for their nuanced and contrasting meanings (Castañer and Oliveira 2020; Keast et al. 2007; McNamara 2012). To overcome this, a prominent concept to characterize different forms of interaction are the so‐called 3Cs of cooperation, coordination, and collaboration. The three notions are commonly conceptualized as a continuum where the intensity and degree of interaction increase from cooperation, over coordination to collaboration (Keast et al. 2007):

1. Cooperation typically involves informal, short‑term relationships where organizations maintain complete autonomy and independent structures, keeping organizational alterations to a minimum (Keast et al. 2007; McNamara 2012). Operating as a low‑risk strategy, it relies on informal channels to share basic information for individual benefit without requiring a unified common goal or deep interpersonal trust (Cigler 2001; Ospina and Yaroni 2003).
2. Coordination refers to more formal relationships with linkages between coordinating hierarchical structures and centralized control and decision‐making. Organizations remain semi‐autonomous as more resources are invested and shared through formal channels to achieve individual goals. Policies, including formal rules, to govern the collective arrangement are developed by supervisory staff as part of an agreed program of action. As such, trust is only necessary between leaders within the hierarchical structure. Potential conflicts tend to be resolved through an external facilitator (Keast et al. 2007; Keast and Mandell 2014; McNamara 2012).
3. Collaboration refers to the most integrated and intense form of interaction (Cigler 2001). Collaborating partners share knowledge, skills, and other resources in order to achieve a common goal and/or tackle a shared problem in a joint approach with high‐levels of interdependence, for example in form of integrated information systems (Cigler 2001; McNamara 2012; Osei‐Kojo et al. 2020). Collaboration requires frequent and open formal and informal communication, participatory decision‐making, jointly developed agreements and rules, a high degree of trust and a leadership style that is not restricted to top‐down direction but encourages all participants to work towards the joint goal (Costumato 2021; McNamara 2012; Osei‐Kojo et al. 2020). Participants ideally work together to resolve or reduce conflicts. For example, conflicts may emerge when participates try to work together while remaining connected to their organizations potentially bringing in divergent goals and needs (Osei‐Kojo et al. 2020). This can lead to a lack of consensus about the meaning of collaboration as well as accountability and power‐sharing issues (Costumato 2021; Osei‐Kojo et al. 2020).

While the literature on public policy and administration has largely taken a “collaborative turn” (Costumato 2021; Osei‐Kojo et al. 2020), some authors argue that “all of the ‘3Cs’ have merit and utility. […] Key to implementing successful integration mechanisms is to decide upfront what results are to be achieved” and see the 3Cs as “complimentary approaches” (Keast et al. 2007, 26, 28).

Since its initiation, authors have variously described and partly criticized the interaction within risk analysis. As described above, separation of RA and RM is likely to facilitate less intense, that is cooperative rather than collaborative forms of interaction (McNamara 2012). The normalized approach to organizing risks has also been associated with a tendency to centralize power and exercise hierarchical oversight and control through standardized plans and protocols (Hardy et al. 2020; Lodge 2009), which aligns, at least in part, with a coordinative approach (McNamara 2012). For Hardy et al. 2020, 1050) controlling practices are most likely to be enacted as risks materialize, especially during crises. However, while controlling may ensure the centralization and standardization needed to deal with anticipated risk incidents, unpredictable aspects of risks, for example during crises but also in the context of emerging or novel risks, can call for improvising with an emphasis on emergent and flexible action and decentralization of authority (Hardy and Maguire 2020; Hardy et al. 2020; Maguire and Hardy 2016; Deverell et al. 2019; Nohrstedt et al. 2018; Hattke and Martin 2020). Similar arguments can be found in the complexity science literature (Costumato 2021; Li et al. 2021). Homer‐Dixon et al. (2022), for example, demand “multi‐level approaches where risk governance is assigned to multiple agencies with overlapping jurisdictions” to deal with increasingly complex, uncertain and ambiguous, that is, “systemic” risks. The risk governance framework (IRGC 2017; Renn 2020; Klinke and Renn 2021) proposes risk analysis as a continuous and iterative process with multiple intersections between understanding and deciding on risk matters, especially for increasingly systemic risks. Specifically, it calls to enable risk assessors and risk managers to develop a common understanding of their tasks and responsibilities through the exchange of risk‐related data, information, and knowledge (ibid). Similarly, North (2020) calls for a “analytic‐deliberative process” that cycles “between learning and analysis and deliberation/evaluation on the adequacy of the analysis and supporting information.” From a structural perspective, these modes of interaction exist along a spectrum that directly impacts functional boundaries. At its baseline, a system can maintain a strict functional separation while operating via required, routine mutual information exchange and light cooperation. However, as interactions shift further up the continuum toward formal coordination and intensive collaboration, the high degree of operational entanglement and shared decision‐making inherently risks blurring or diminishing the functional separation between the independent scientific assessment and the political management phases.

Particularly for collaborative (Hardy et al. 2005) but also cooperative (Thompson and Bloom 2000) forms of interaction, communication is often seen as central (see also North 2020). Arguably, risk managers need to understand RA results in order to evaluate the risks associated with different RM options as well as ongoing research, areas of uncertainties and potential research needs. This necessitates competencies and close communication between risk assessors and risk managers (Thompson and Bloom 2000; Vareman and Persson 2010). Studying the role of communication between environmental risk assessors and managers, Thompson and Bloom (2000, 348) suggest for risk assessors to “consider the larger context,” “present thorough risk characterizations,” and “be prepared to discuss the differences in risks associated with the management options.” Especially in the early stages of the risk analysis process, many argue that “having more and more effective dialogue between risk assessors and risk managers is the only way to provide a better basis for management and value relevant risk assessment” (Calow 2014, 689). For Robinson and Levy (2011, 1343), “in the formulation and problem formulation and scoping phase, risk assessors should consider several generic concerns, including generating the basic information needed to define potentially feasible and effective options (taking into account legal, political, and resource constraints) and determining how to best provide the data needed for valuation of the range of potentially significant health outcomes.” Beyond that, they call for “spirals, with several loops of data collection, analysis, and evaluation that are reiterated as needed given the results of the previous steps and information needed by decision makers‐subject to time, resources and other constraints.” Crucially, a frequent omission in these early framing and scoping discussions is a forward‐looking evaluation of external complexities, such as the potential shifts in global trading partnerships and the rapid evolution of new technological landscapes. A primary contemporary example is the integration of artificial intelligence (AI) systems. AI technologies are increasingly shifting paradigms in both RA and RM, where algorithms assist in monitoring compliance and weighing policy alternatives. Failing to anticipate these technological and trade disruptions during early RA–RM dialogues can result in rigid, rapidly obsolete regulatory frameworks.

Indeed, many argue that interaction between RA and RM has always been both intended and implemented, especially in the food context (Ely et al. 2009, 21; see also Rimkutė 2015). From the outset of the risk‐analysis process—when defining the problem, establishing the scope of RA, and setting policy and framing conditions—interaction between RA and RM are necessary and inevitable (Ely et al. 2009; Millstone 2009; Stirling and Scoones 2009). This operational reality is entirely undisputed in international food safety governance and has been structurally embedded within the foundational Codex Alimentarius guidance for 25 years. Rather than viewing communication as an unscripted compromise to absolute separation, international frameworks (such as Codex Alimentarius) have long recognized that functional separation explicitly relies upon a structured, multi‐directional interface to operationalize scientific independence effectively. Similar statements can be found in other fields. Vareman and Persson (2010, 689), for example, speak of a “risk formulation privilege” on the side of the risk manager that “influences both what assessments are to be performed and their degree of certainty.” Looking at scientific experts' view on their role as policy advisors in the context of electromagnetic fields, Spruijt et al. 2019, 9) found “support for the view that there is no strict separation between science and policymakers, but rather that the two are interlinked.” For Greenberg et al. 2015, 1966) “Recommendation 1 (clear conceptual distinction between risk assessment and management) has proven difficult to sustain, especially given the reality that risk management decisions sometimes drive risk assessment and there is now earlier and more dynamic feedback between risk assessors and risk managers”. However, Spruijt et al. 2019, 972) found “marked differences in views about whether and how experts should interact with policymakers” (Spruijt et al. 2019, 972; see also Spruijt et al. 2013).

Within sociology of science and science and technology studies, authors have long discussed the inherent links between regulatory science and policy making and the different, sometimes contrasting, views on that link (see e.g., Jasanoff 1994; Demortian 2017, 2023). For Demortian (2023, 408) “regulatory science emerges from scientists' close and enduring cooperation with institutional and policy actors to forge agreed‐upon rules of demonstrating and calculating the existence of risks.” However, “STS [Science and Technology] research has shown that giving scientific advice confronts professional scientists with complicated dilemmas regarding the value of applied research in their scientific field and the time they devote to it, how to balance the defense of neutral facts and the tailoring of knowledge to policy needs, and how to defend the value of science to address complex questions while accepting the limits and uncertainties of available knowledge” (Demortain 2023, 409). Studying researchers engagement in scientific advice in a toxicology laboratory they found “a variety of moral economies motivate engagement and tensions in the social organization of research [‥] sparked by the networks through which scientists get coopted and socialized into scientific advice” (Demortian 2023, 425) This resonates with the wider sociology of science literature which sheds light on the complex relationship between the “two different systems” of science and politics (Sundqvis and Linke 2024; see also Weingart et al. 2000). Studying the IPCC Sundqvis and Linke (2024), for example conclude “IPCC's self‐imposed ambition to keep a clear distance from political negotiations never gives a true presentation of the actual situation, since there is ongoing cooperation between scientists and government representatives. Nevertheless, this ambition of separation is used strategically to survive in a hostile world of strong fossil fuel interests. Sticking to ‘pure science’ is both a response to this situation and an example of how a division of labour between separate worlds is the result of processes of co‐production” (Sundqvist and Linke 2024). To summarize, across communities of research and practice we find disparity between stipulated autonomy and calls for and partial implementation of horizontal integration of risk analysis tasks through different forms of interaction. However, few studies have looked at how this potential tension affects current interaction and overall risk analysis. Indeed, we generally find very little research on how those involved in RA and RM currently perceive their mutual interactions (Bier 2001; Thompson and Bloom 2000), to what extent these perceived practices align with the interaction types cooperation, coordination, and collaboration, and what barriers may exist for closer forms of interaction. In evaluating more holistic frameworks of risk analysis, scholars like Schweizer (2021, 91) argue that there is an apparent demand for research investigating novel processes of coordination and cooperation that move beyond the boundaries of classic top‐down intervention. More generally, and beyond the risk analysis context, the dynamics and performance of collaborative but arguably also cooperative and coordinative interaction has received little scholarly attention (Costumato 2021). In the following, we will explain how this study used survey research to address these gaps.

## 3 Method

### 3.1 Participants

Our study focused on professionals working on the assessment and management of food and feed risks in EU member or candidate states. An initial sample of 120 participants completed the survey; however, 21% of the questionnaires were excluded due to insufficient data. Therefore, the final sample comprised 95 participants. The participants' average age was 50 years (SD = 9.2), with an average of 14 years (SD = 8.1) of experience in risk analysis. Regarding gender, 48% identified as female, 44% as male, and 8% preferred not to respond.

Participants represented 23 identified EU countries, while 10 respondents opted not to disclose their specific country of origin. The number of responses per country ranged from 1 to 17 (median = 2, mean = 3.7), with approximately one‐third of the responses originating from Germany, Ireland, and Italy. To ensure respondents were confident that they could not be identified, no further information regarding specific institutional affiliation was collected beyond country representation.

Regarding their professional roles, 55 individuals (58%) worked exclusively in RA, 31 individuals (33%) worked exclusively in RM, and 9 individuals (9%) reported performing both RA and RM functions. To maintain analytical clarity, these nine dual‐role participants were excluded strictly from the comparative analyses between separate RA and RM cohorts (e.g., the MANOVA and Chi‐Square tests of independence) because their boundary‐spanning positions would conflate discrete group contrasts. However, they were not removed from the study entirely; their responses were fully retained and included in all pooled sample analyses, including the K‐means clustering and the multinomial logistic regression models, to ensure their systemic expertise was captured in the broader evaluation of risk analysis quality.

### 3.2 Instruments

The questionnaire comprised three sections: background questions, questions on the last risk analysis, and questions on general challenges and areas for improvements. The purpose of the background questions was to better understand the context of the risks analysis (e.g. routine vs. rapid risk analysis) and to differentiate between individuals that describe themselves as only working in RA or RM and those who identify themselves as performing both RA and RM. Based on the identification of these risk analysis types, filters were applied to relevant sets of questions. For a comprehensive overview of the questionnaire structure, please refer to Figure 1. If participants indicated that they performed both RA and RM in their last analysis, they did not receive any questions regarding their interaction with RM or RA. Instead, they received another set of questions that is beyond the scope of this publication.

**FIGURE 1:**
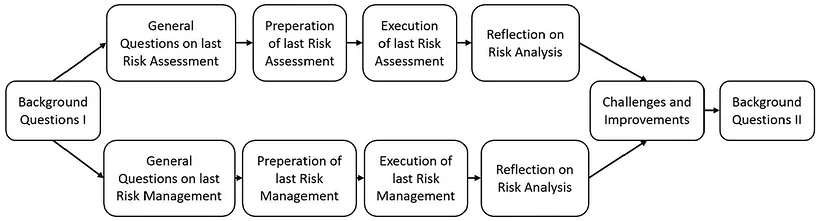
Structure of the questionnaire.

The subsequent questions were categorized into general aspects, preparation, execution, and reflection. By focusing the questionnaire on the participant's most recent risk analysis, we aimed to elicit specific and recent memories, addressing the challenge of providing a concise response. This approach allowed us to obtain a more detailed and vivid understanding of the interaction.

#### 3.2.1 Interaction Assessment Using the 3C Framework

The main part of the survey assessed interaction between RA and RM based on the cooperation, coordination, and collaboration (3C) framework developed by McNamara (2012). We operationalized 10 interaction dimensions: decision making, organizational design, formality of agreement, information sharing, key personnel, organizational autonomy, resource allocation, systems thinking, trust, and turf issues. To measure these concepts accurately, five of the dimensions were assessed using a single multiple‐choice question, while the remaining five dimensions utilized a two‐question sequential structure, resulting in a total of 15 survey items. Specifically, organizational design, formality of agreement, turf issues, resource allocation, and systems thinking were operationalized as two‐step sequential or multi‐part items.

Each of the 10 dimensions was assessed with one or two questions, resulting in a total of 15 items. The two‐item dimensions (Formality of Agreement, Turf Issues, Resource Allocation) were realized with a sequential skip‐logic meaning that they were measured by a two‐step sequence. For example the “Formality of Agreement” dimension consists of the two questions “To what extent was your interaction with RA / RM formalized?” and “Who sets these informal agreements? In that case the skip logic means that only if the first question is answered with ʻʻThere were informal (e.g., verbal) agreements on how our communication with risk assessment should proceed” the second question was meaningful. These two‐item dimensions were not intended to form a reflective scale. As a result, standard inter‐item correlation or Cronbach's *α* are not applicable for these formative dimensions.

Questions were multiple‐choice, with options reflecting the characteristics of cooperation, coordination, or collaboration as defined by McNamara (2012). The level of measurement was nominal. For example for the dimension “Information Sharing” participants were asked, “Besides the official mandate and the delivery of the risk assessment report, how would you characterize your communication with risk management?” Options included:

- “We communicated only if needed on a spontaneous basis.” (Cooperation)
- “We had formal proceedings for further information exchange.” (Coordination)
- “We communicated openly and frequently.” (Collaboration)

The interaction style in bracket after each option was not shown in the questionnaire.

Table 1 provides an overview of how we translated the ideals described by McNamara (2012) within each dimension into survey questions. Most 3C questions were single choice questions, except for the dimension information sharing, where several modes of information sharing may occur within the same risk analysis process. The order of all items was displayed randomly to account for primacy and recency effects.

**TABLE 1:** Overview of survey questions.

Dimension; remarks on weighting	Questions	Cooperation	Coordination	Collaboration
Organizational Design, count is solely based on second question	“Which parts of the risk analysis process is the organization for which you work involved in?”	… only provides risk assessment.ʼʼ OR ʻʻ… only provides risk management.		“… provides both risk assessment and risk management.”
	“Is there a person or entity who coordinates your interaction with your risk analysis counterpart?”	“No” OR “I don't know / I prefer not to say.”(Indicates the absence of an active, centralized intermediary shaping daily isolated workflows)	“Yes” (Reflects a dedicated, centralized hierarchical coordinating entity)	“No” OR “I don't know / I prefer not to say.” (Indicates that peer‐to‐peer interaction occurs directly without the reliance on a bureaucratic middleman)
Formality of agreement; each question counts 0.5	“To what extent was your interaction with RA / RM formalized?”	“There were informal (e.g. verbal) agreements on how our communication with risk management should proceed (e.g., in terms of roles and responsibilities).”	“There were specific formal guidelines for our communication with risk management (e.g., in terms of roles and responsibilities).”	“There were informal (e.g. verbal) agreements (…)” AND “There were specific formal guidelines (…)”
	“Who set these informal agreements?”	“The agreements were set by either risk assessment or risk management.”	“The agreements were set by a coordinating entity.”	“The agreements were set jointly.”
Organizational autonomy	“During the last risk analysis, to what extent did you work autonomously from RA / RM?”	“The risk assessment was performed fully autonomously.”	“A coordinating entity from my own organization coordinated the risk analysis process.” OR “A coordinating entity from another organization coordinated the risk analysis process.”	“I worked jointly with risk management / assessment.”
Key personnel	“Which of the following scenarios best describes your interaction with risk management during the last risk analysis?”	“I interacted directly with risk management on a working level, supervisory staff was only involved during approval processes.”	“A coordinating entity facilitated my interaction with risk management.”	“Me, supervisory staff and potential colleagues interacted on equal footing with risk management / assessment.”
Info sharing	“Besides the official mandate and the delivery of the risk assessment report, how would you characterize your communication with risk management?”	“We communicated only if needed (e.g., additional questions) on a spontaneous basis.”	“We had formal proceedings for further information exchange (e.g., regular meetings).”	“We communicated openly and frequently.”
Decision making	“Who made the decisions regarding the risk assessment methodology during the last risk analysis?”	“Risk assessment alone” OR “Risk management alone”	“A coordinating entity”	“Risk assessment and risk management collectively”
Turf issues**;** for cooperation OR is possible; for coordination and collaboration only combination counts as 1	“Did you experience conflicts regarding responsibilities and decision making power between risk assessment and risk management during the last risk analysis?”	“No”	“Yes”	“Yes”
	“How did you mainly deal with these conflicts?”	“We did not address the conflicts.”	“We resolved the conflicts with help of a third party.”	“We resolved the conflicts in a team effort without the involvement of a third party.”
Resource Allocation; combination counts as 1	During the last risk analysis, did you share any resources (i.e., budget, personnel, facilities…) with risk management?	“No”	“Yes”	“Yes”
	“During the last risk analysis, to what extent did you work autonomously from risk management?”		“The risk assessment was performed fully autonomously. OR A coordinating entity from my own organization coordinated the risk analysis process. OR A coordinating entity from another organization coordinated the risk analysis process.”	“I worked jointly with risk management / assessment”
Trust	“Which role did trust play for the success of the last risk analysis?”	“It was not necessary that risk assessors and risk managers trusted each other.”	“Trust was only necessary between supervisory staff of risk assessment and risk management, not on a working level.”	“It was crucial that all partners trusted each other.”
Systems thinking	“Did you use shared risk analysis information systems (e.g., databases or knowledge platforms) with risk management / risk assessment?”	“No”	“Yes”	“Yes”
	“Which role did these shared information systems play?”		“They were only used if they facilitated the risk assessment.”	“They were used to facilitate the overall risk analysis process.”

Responses were coded according to the 3C categories, assigning a score for cooperation, coordination, or collaboration for each dimension. Each dimension contributed equally to the overall interaction profile. This way we were able to gain insights into interaction forms based on the overall composition of shares of the different 3C dimensions. Because the 3C dimensions are formative traditional indices of internal consistency (e.g., Cronbach's *α*) are not applicable. Instead, we ensured face validity via McNamara's (2012) framework and by pretesting the questionnaire with external risk assessors and risk managers.

Outcome measurement: The perceived quality of risk analysis was self‐assessed by rating the effectiveness (“meeting risk analysis goals”) and efficiency (“minimizing resource requirements”) of the last risk analysis on a five‐point Likert scale (1 = *very poor*, 5 = *very good*). These two items were aggregated by mean to create a composite quality score. The reliability analysis yielded a Cronbach's *α* of 0.90, indicating high internal consistency.

### 3.3 Procedure

The survey took place within the EFSA partnering‐grant project ENhanced COMmunication in Risk Analysis (ENCOMRAN). The population of interest consisted of individuals working on the assessment and management of food and feed risks in EU member or candidate states. Specifically, this involved risk assessors connected to the EFSA—an independent EU RA body—and risk managers representing governmental bodies across Europe. (*Note*: In the United States, the Environmental Protection Agency [EPA] and the Food and Drug Administration [FDA] are responsible for both RA and RM. While housed within the same agencies, these functions are treated separately with distinct staff categories, functionally similar to the European model). The survey was developed iteratively by the ENCOMRAN consortium—comprising RA, RM, and risk‐communication practitioners from four EU Member States—and then pre‐tested and refined with external risk managers and risk assessors.

Data collection occurred from July to October 2022. The finalized survey was administered online using a secure platform. Participants were recruited through both formal and informal channels:

- *Risk Assessors*: Contact information was obtained via the EFSA focal point network.
- *Risk Managers*: Invitations were disseminated through the Heads of Food Safety Agencies (HoA) network.

An invitation letter explaining the study's purpose and ensuring confidentiality was sent to these primary contacts, with instructions to forward the anonymized online survey link to relevant colleagues. Follow‐up reminders were sent to increase participation, and personal contacts were utilized in countries that initially yielded no responses. All participants provided informed consent prior to beginning the questionnaire. Because the survey was distributed through these organizational networks and intermediaries, the exact number of ultimate recipients is unknown, precluding the calculation of a traditional response rate. Consequently, our analysis focuses on the quality, internal validity, and broad geographical representation of the 95 finalized responses, which capture a diverse cross‐section of perspectives across the EU.

### 3.4 Data Analysis

We analyzed the data using R 4.4.1 (with packages tidyverse, stats, car, factoextra, cluster, nnet) for descriptive statistics, variance analysis, correlation analysis, clustering, and multinominal logistic regression. Research Question 1 (“What is the current state of interaction between risk assessors and risk managers?”) was addressed by descriptive statistics in order to obtain a general impression regarding the interaction between RA and RM. RQ1a (“How does the perceived interaction between risk assessors and risk managers vary?”) was addressed with a multivariate variance analysis, specifically a MANOVA was calculated in order to compare those identifying themselves as risk assessors and those identifying themselves as risk managers in terms of their interaction score. A chi‐square test of independence was used to determine differences among RA, RM for the set of quality outcome variables. We also used MANOVA to analyze if and how interaction forms differ between routine risk analysis and rapid risk analysis. RQ1b: “Are forms of interaction correlated with perceived quality of risk analysis?” is analyzed using Pearsons correlation coefficient.

The second RQ “Can the risk analysis process be improved by specific forms of interaction?” with its subquestion RQ2a “Which form of interaction positively affects the perceived quality of risk analysis” was addressed using K‐means clustering to create distinct clusters of interaction forms throughout the risk analysis process. Subsequently, differences across these clusters with regard to the perceived quality of the risk analysis process were assessed using the Kruskal−Wallis test.

To answer RQ2b “Which underlying interactional factors have an impact on the perceived quality of risk analysis.” a multinomial logistic regression model was employed with the dependent variable “quality.” This model was chosen because the dependent variable was categorical. The independent variables comprised the 3C dimensions. Any missing data were imputed using the mean value of the valid data. Across the entire dataset matrix used for the regression model (86 participants ×10 interaction dimensions = 860 data points), mean imputation was required for only five entries (0.58% of the total matrix), representing an exceptionally low missingness rate. The results of the variance inflation factor (VIF) indicated no issues of multicollinearity among the independent variables. To execute the multinomial model, participants were categorized based on their respective responses to the dependent variable. The “low” group included participants with scores of 1, 2, or 3, while the “moderate” group encompassed those with a score of 4, and the “high” group consisted of participants scoring 5 on the quality scale. While this categorization reduced the granularity of the information, less than 5% of participants provided negative responses on the quality item. The reference groups for the dependent variables were the “high” groups, and for the independent variables, they were the “collaboration” groups.

## 4 Results

### 4.1 Context of Last Risk Analysis

The baseline characteristics of the evaluated risk analysis files—encompassing hazard subject areas, operational categories, and baseline timelines—are detailed in Tables 2, 3, 4. Because respondents reported independently on their most recent individual files via network‐wide snowball sampling, the dataset represents aggregate cross‐sectional workflows rather than linked case‐specific dyads. While the sample reflects a multi‐hazard portfolio with strong proportional alignment between the two cohorts across biological and chemical domains (Table 2), their operational profiles diverge structurally. Risk assessors predominantly focused on routine RAs (56%) spanning longer timeframes, whereas risk managers handled a more evenly distributed portfolio of expert opinions and rapid assessments (38%), with a majority of files completed in less than a month (Tables 3 and 4).

**TABLE 2:** Subject area of the last risk analysis.

Subject area	RA (*n* = 55)	RM (*n* = 31)
Biological hazards in drinking water.	1	
Biological hazards in feed.	3	2
Biological hazards in food.	22	8
Biological hazards in water to animals.	2	
Chemical hazards in drinking water.	3	3
Chemical hazards in feed.	4	4
Chemical hazards in food.	31	13
Chemical hazards in water to animals.	2	1
Emerging risks (related to food or feed).	5	3
Other (please specify):	5	7
Physical hazards in feed.	1	1
Physical hazards in food.	1	3

*Note*: On concordance: Due to the anonymized cross‐national survey design, responses reflect independent individual files rather than paired, case‐specific RA‐RM dyads. Aggregate sample proportions; however, confirm balanced thematic representation across biological and chemical domains.

**TABLE 3:** Categories of the last risk analysis.

Category	RA (*n* = 55)	RM (*n* = 29)
Expert opinion, i.e., a summary of evidence‐based views given by experts in the field (often used when there is a lack of data).	9 (16%)	6 (21%)
Rapid risk assessment, i.e., a risk assessment based on limited data due to time constraints (often used in initial stages of an event or after an incident of potential public health concern).	12 (22%)	11 (38%)
Routine risk assessment, i.e., a systematic review of all available scientific data to evaluate the magnitude of risks associated with certain hazards.	31 (56%)	9 (31%)
Other:	3 (5%)	3 (10%)

**TABLE 4:** Length of the last risk analysis.

Length	RA (*n* = 55)	RM (*n* = 28)
Less than a month	18 (33%)	16 (57%)
1–3 months	11 (20%)	5 (18%)
4–6 months	11 (20%)	2 (7%)
7–12 months	5 (9%)	1 (4%)
More than a year	10 (18%)	4 (14%)

The majority of risk analysis was categorized as routine risk analysis (47.6%) compared to rapid risk analysis, expert opinion and option “other”. A MANOVA was used to determine whether there are significant differences between routine risk analysis and rapid risk analysis regarding cooperation, coordination, and collaboration. We tested the assumptions of a MANOVA, including multivariate normality, homogeneity of variance‐covariance matrices, and linearity, and found no violations. The MANOVA revealed a significant effect of routine risk analysis and rapid risk analysis on the dependent variables. The Pillai's Trace test statistics is statistically significant (Pillai's trace = 0.15, *F*(3, 59) = 3.38, *p*  = 0.024). A follow‐up univariate ANOVA showed that the collaboration score is significantly different (*p* < 0.01) between routine risk analysis and rapid risk analysis. In other words, risk analysts collaborate more during rapid risk analysis than they do during routine risk analysis. No significant differences were found for cooperation and coordination.

### 4.2 What Is the Current State of Interaction Between Risk Assessors and Risk Managers? (RQ1)

The sum of scores of all 3C questions resulted in 57% cooperation, 17% coordination, and 26% collaboration across all dimensions. As shown in Figure 2 RA had a higher cooperation score than RM (RA: 65%; RM 42%). RM had higher collaboration (RA: 20%; RM 36%) and slightly higher coordination values (RA: 15%; RM 22%). The statistical significance of these descriptive differences is evaluated in the subsequent section with answering Research Questions 1a.

**FIGURE 2:**
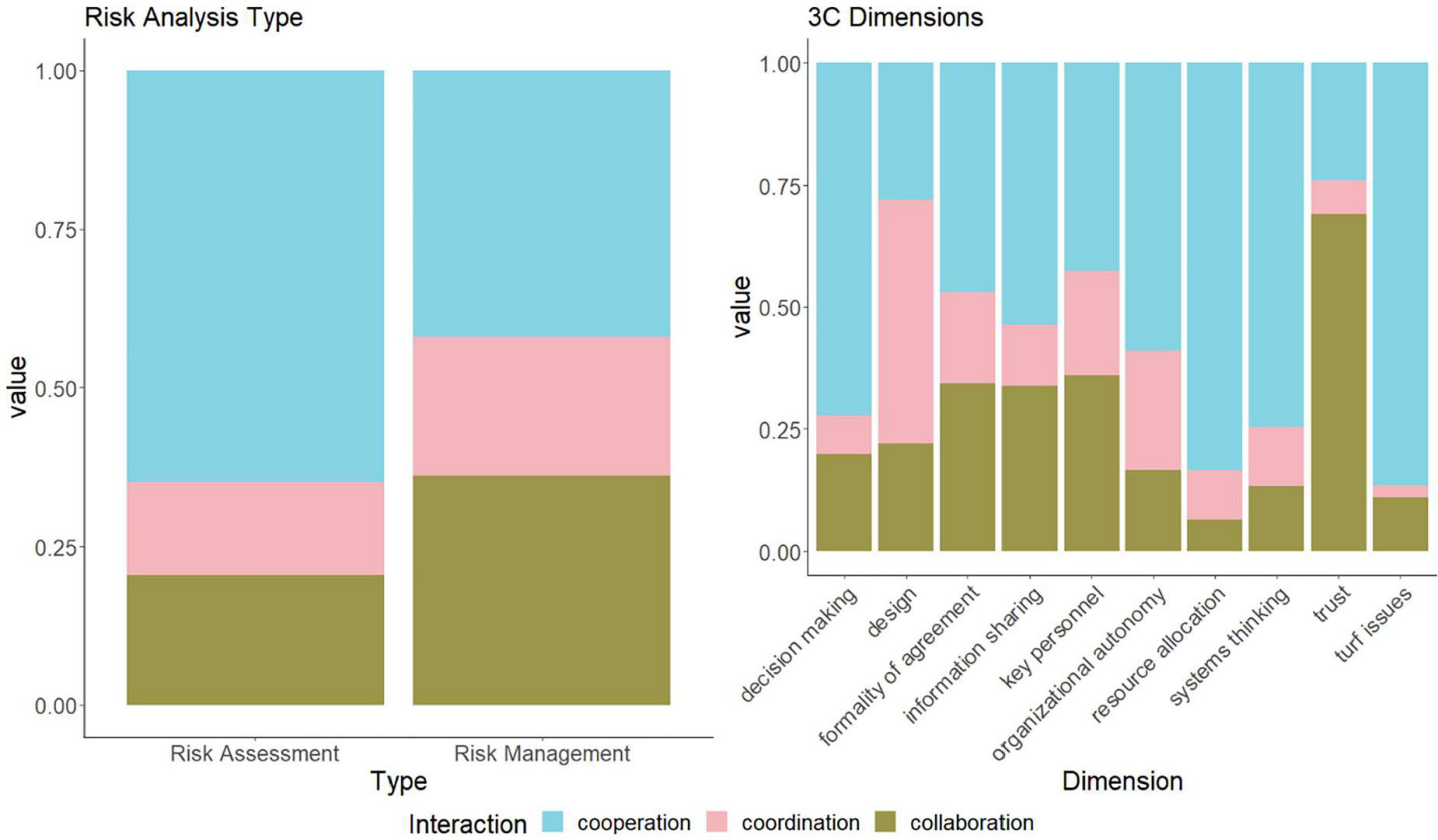
This is Fig 2 Left hand side, Distribution of the 3C score for risk assessment and risk management; Figure 3 Right hand side, Distribution of the 3C score across 3C dimensions.

Figure 3 indicates that for most dimensions, overall interaction aligned strongest with cooperation (especially decision‐making, resource allocation, systems thinking, and turf issues). This is except for the dimension organizational design, where respondents most dominantly described a coordinative organizational setting. In terms of trust, most respondents stated that it was crucial that all partners trusted each other, which aligns with collaboration.

To examine the relationship between the three interaction modes, we calculated composite scores for each participant based on their responses across all 10 dimensions. Table 5 presents the Pearson correlation coefficients between these three composite scores, representing how the prevalence of one interaction mode relates to the others within the sampled population. Cooperation is moderately and negatively associated with both Coordination (*r* = −0.54) and Collaboration (*r* = −0.49), while Coordination and Collaboration show a negligible correlation (*r* = 0.05).

**TABLE 5:** Pearson correlation coefficients between the composite scores for cooperation, coordination, and collaboration (*n* = 86).

	Cooperation	Coordination	Collaboration
Cooperation	—	−0.54	−0.49
Coordination	−0.54	—	0.05
Collaboration	−0.49	0.05	—

### 4.3 How Does the Perceived Interaction Between Risk Assessors and Risk Managers Vary? (RQ1a)

We used a MANOVA to determine whether there are significant differences between RA and RM on the dependent variables cooperation, coordination, and collaboration based on the 3C score as described above. We tested the assumptions of MANOVA, including multivariate normality, homogeneity of variance–covariance matrices, and linearity, and found no violations. The MANOVA revealed a significant effect of RA and RM on the dependent variables. The Pillai's trace test statistics is statistically significant (Pillai's trace = 0.26, *F*(3, 82) = 9.95, *p* < 0.001) and indicates that risk managers and risk assessors differ statistically significant in the perceived form of their interaction.

We then conducted follow‐up univariate ANOVAs to determine which interaction scores were driving the significant effect. The results of this analysis show significant differences between RA and RM in terms of cooperation (*p* < 0.001) and collaboration (*p* < 0.001), while there is no significant difference regarding coordination (Figure 4).

**FIGURE 4:**
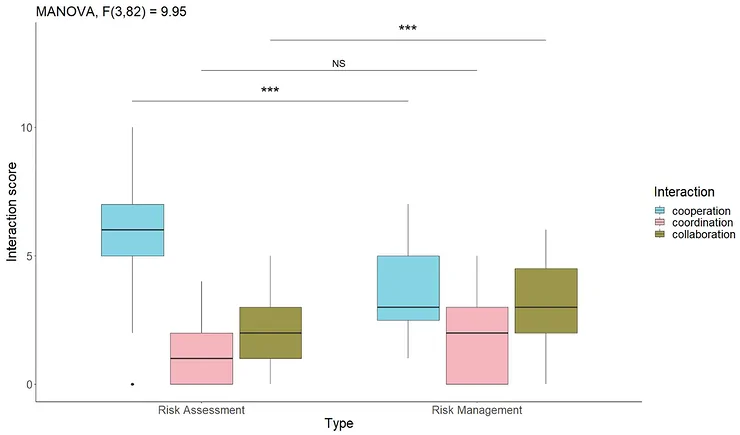
Differences of risk analysis type and interaction score (***p < 0.001, and NS indicates no statistical significance).

Different perceptions of the interaction between risk assessors and risk managers become particularly apparent when isolating one of the 10 interaction dimensions: the autonomy dimension. Here most risk assessors indicated that their last risk analysis was performed fully autonomously (72.2%), while the view of risk managers was more diverse (see Table 6).

**TABLE 6:** Autonomy during the last risk analysis.

Autonomy	RA (*n* = 54)	RM (*n* = 29)
A coordinating entity from another organization coordinated the risk analysis process.	2 (4%)	2 (7%)
A coordinating entity from my own organization coordinated the risk analysis process.	5 (9%)	10 (35%)
I worked jointly with risk management.	6 (11%)	7 (24%)
The risk assessment was performed fully autonomously.	39 (72%)	7 (24%)
Other	2 (4%)	3 (10%)

To further explore this finding, we performed a chi‐square test of independence to determine differences between RA and RM with regard to the autonomy variable. There is a significant difference between RA and RM: *X* ^2^ (1, *N* = 81) = 14.7, *p* = 0.001. All expected cell frequencies exceeded 5, so the chi‐square assumptions were met. In other words, RA are more likely than RM to perceive their work as fully autonomous.

### 4.4 Are Forms of Interaction Correlated With Perceived Quality of Risk Analysis? (RQ1b)

Table 7 presents the strength and direction of the linear relationship between quality (mean of “effectiveness” and “efficiency”) and the “cooperation,” “coordination,” and “collaboration” scores. There's a mild positive relationship between “quality” and “cooperation” (*r* = 0.24), a mild negative relationship with “coordination” (*r* = −0.25), and no relationship with “collaboration” (*r* = 0.08).

**TABLE 7:** Correlation of perceived quality and 3C scores.

	Pearson's correlation coefficient
Quality and cooperation	0.24^*^
Quality and coordination	−0.25^*^
Quality and collaboration	0.08

^*^ *p* < 0.05.

### 4.5 Which Form of Interaction Positively Affects the Perceived Quality of Risk Analysis? (RQ2a)

The K‐means clustering (explaining 54.8% of the total variance) identified three distinct typologies of real‐world interaction: Cluster 1 (“Coordinated”, *n* = 22) characterized by high coordination and low cooperation; Cluster 2 (“Cooperative”, *n* = 35) characterized by high cooperation but low collaboration and coordination; and Cluster 3 (“Collaborative”, *n* = 29) characterized by high collaboration.[Table risa70376-tbl-0008]

**TABLE 8 risa70376-tbl-0008:** Cluster means of the normalized data.

Cluster	Cooperation	Coordination	Collaboration
1	−1.06	1.29	0.02
2	0.82	−0.43	−0.74
3	−0.19	−0.46	0.88

When assessing the perceived quality of risk analysis across these three clusters, the Kruskal−Wallis test indicated no statistically significant difference. While this appears as a null finding, it offers a vital empirical lesson for public administrators: it demonstrates that no single interaction typology is universally superior, directly challenging the prevailing “collaborative turn” in public management literature that uniformly demands deeper collaboration. Because structurally coordinated, informally cooperative, and highly collaborative frameworks all achieve statistically identical, high‐quality outcomes, the key takeaway is that organizations have multiple viable pathways to success. Rather than expending resources forcing an intensive collaborative model onto every routine file—which introduces heavy bureaucratic overhead—practitioners can trust that lighter cooperative or structured coordinative models are equally capable of delivering highly effective and efficient risk analyses depending on the task's context. We performed K‐means clustering on the pooled sample to identify overall, cross‐role interaction typologies. Because we assured participants that no individual or agency could be identified, affiliation and country data were anonymized. As a result, we cannot report cluster membership by specific country or agency—instead, we confirm that no single nation or organization dominated any cluster.

### 4.6 Which Underlying Interactional Factors Have an Impact on the Perceived Quality of Risk Analysis. (RQ2b)

The multinomial logistic regression model tested the impact of different 3C dimensions on perceived quality. The model fit information was *χ* ^2^(40) = 65.1, *p* = 0.007. The information about the fit quality of the model were Pearson's chi‐square test *χ* ^2^(104) = 98.0, *p* = 0.648. Nagelkerke's pseudo *R* ^2^ was 0.652. Table A1 in the Appendix shows the results of the model's regression coefficient, while Table A2 presents the regression coefficients for the two item dimensions. There was only one significant result: the variable “information sharing” had a coefficient that was statistically different from zero at the level of *p* < 0.05. Respondents in the “information sharing—coordination” condition (i.e. formal proceedings for further information exchange) have a lower probability to score higher in the high‐quality category compared to respondents in the “information sharing—collaboration” condition (i.e. open and frequent communication). Although our subsample (*n* = 86 valid responses for this model) provided adequate power for the multinomial logistic model with 20 predictors (≈ 4.30 cases per predictor), larger samples would yield more stable coefficient estimates. To preserve the exploratory integrity of the 3C dimensions and prevent spurious, sample‐dependent predictors, we avoided post hoc stepwise selection. Stepwise approaches risk capitalizing on random sampling variation, artificially inflating Type I error rates, and biasing regression coefficients upward. We therefore restrict our analysis strictly to the conservative simultaneous model architecture.

## 5 Discussion

This study mapped the current state of interaction as perceived by those working on food and feed RA and RM tasks across Europe. Our results show that current interaction tends to be a highly individual composition of shares of the 3Cs but overall aligns strongest with cooperation. This was strongest in the dimensions decision‐making, resource allocation, systems thinking, and turf issues. In other words, most respondents reported fully separated decision‐making processes, no use of shared information systems or other resource sharing and the absence of conflicts or open address of conflicts. Only in terms of organizational design, respondents most dominantly described a person or entity coordinating the risk analysis process. For the trust dimension, most respondents stated that it was crucial that all partners trusted each other. This high baseline requirement aligns precisely with McNamara's (2012) framework, which positions multi‐level, systemic trust as a key characteristic of collaborative environments where structural boundaries are highly permeable. To further refine this interpretation, future research should explore more granular modalities of trust across the interaction spectrum. While the 3C framework successfully categorizes trust based on its systemic and organizational scale, practitioners note that localized trust remains essential even within strictly separated cooperative or coordinative structures. For instance, low‐interaction cooperative models require actors to rely implicitly on the independent scientific competence of their counterparts without continuous oversight. Thus, future work would benefit from developing multi‐layered sub‐metrics—distinguishing between systemic institutional trust and localized technical trust—to capture these subtle working‐level dynamics without altering the broader structural indices of the 3C framework.

Moreover, we compared routine risk analysis and rapid risk analysis with regard to different forms of interaction. We found that rapid risk analysis tends to be more collaborative than routine risk analysis. This finding contributes to discussions on preferred forms of interaction of public administration in crises situations (Hardy et al. 2020; Deverell et al. 2019; Nohrstedt et al. 2018, Hattke and Martin 2020). Given that most of the participants identified their risk analysis work as routine; however, there is a clear need for future research aimed at the interplay of different forms of interaction throughout the “risk cycle” (Hardy et al. 2020).

The intercorrelations among 3C scores (Table 5) further underscore that cooperation, coordination and collaboration operate as distinct—and in some cases opposing—modes of interaction in EU food‐and‐feed risk analysis. The moderately negative associations between Cooperation and both Coordination (*r* = –0.54) and Collaboration (*r* = –0.49) suggest that episodes characterized by informal, need‐based exchanges are unlikely to incorporate formalized control structures or the high interdependence of collaborative efforts. By contrast, coordination and collaboration are essentially uncorrelated (*r* = 0.05), indicating that formal linkage mechanisms (e.g., agreed procedures, supervisory oversight) can exist independently of the more intensive, trust‐based information sharing typical of collaboration. In practical terms, these patterns imply that when agencies seek to strengthen one mode of interaction, for example, by introducing more structured coordination they should not assume this will automatically foster deeper collaboration.

Table 7 shows that perceived quality of the risk analysis process correlates positively, albeit modestly, with cooperation (*r* = 0.24), negatively with coordination (*r* = –0.25), and not at all with collaboration (*r* = 0.08). In practical terms, when assessors and managers engage in informal, need‐based exchanges (cooperation), they tend to judge the process as more effective and efficient. However, this preference for light‐touch, spontaneous cooperation must be interpreted within the broader temporal lifecycle of food safety policy. The viability of rapid, informal information exchange during a live risk analysis is fundamentally predicated on a long‑term foundation; government agencies often begin conducting or funding targeted scientific research up to ten years prior to the initiation of formal mandates. It is this decade‐long accumulation of robust, pre‐existing data that equips both assessors and managers with the shared baseline knowledge necessary to make informal cooperation effective. Without this deep historical investment in data generation, light‐touch interactions would likely prove insufficient for establishing scientifically sound RM approaches. It also underscores that calls for “more collaboration” should be targeted: only those dimensions of interaction that add genuine value (here, rapid, adaptive cooperation) will translate into higher perceived quality.

In search for interaction patterns which relate to or affect perceived quality of risk analysis we conducted K‐means clustering and regressions analysis. We found a mild positive relationship between quality and “cooperation,”, a mild negative relationship with “coordination,” and no relationship with “collaboration.” When looking at individual 3C dimensions, we found strong evidence that coordinative information sharing has lower probabilities to produce a higher “quality” score than collaborative information sharing. In other words, those that stated they would communicate openly and frequently are more likely to have positive perceptions of the risk analysis efficiency and effectiveness. This supports encouragements of risk assessors and risk managers to engage in closer dialogue during the risk analysis process (Calow 2014; IRGC 2017; Thompson and Bloom 2000; Vareman and Persson 2010; Robinson and Levy 2011).

Overall, this strong statistical preference for collaboration only holds true for 1 of 10 dimensions. Other than that, the results of correlation, clustering, and regression analysis do not support strong generalized calls for more collaboration or cooperation. Specifically, the cluster analysis revealed three highly distinct interaction typologies (structurally coordinated, informal cooperative, and highly collaborative), yet highly collaborative interactions did not yield significantly higher perceived quality than the others. Rather than an undirected call for more collaboration or cooperation, this prioritizes a differentiated view onto the topic, where risk analysis is context dependent and nuanced, that is constituted of an individual composition of shares of the 3Cs. As organizations operating in the human services arena, which arguably also applies to those working in the risk field, are likely to be confronted by changing circumstances and requirements, they will benefit from continually monitoring the operating environment and adjusting the nature and strength of interactions (Keast et al. 2007). In doing so, they may include an acknowledgement and reflection on the systematic differences in perception and communication between the systems of science and politics (Weingart et al. 2000). Relatedly, Hardy and Phillips (1998) remind to consider potential power dynamics. For them, collaboration may be easier when power is widely distributed. Otherwise, collaboration, and potentially other forms of interaction, may be used to protect or improve the position of the powerful organization and disadvantage less powerful through cooptation or exclusion. While questions of power and their influence on different forms of interaction have been outside the scope of this research, they certainly remain a relevant area for future research.

There were significant differences in perception of interaction within the same risk analysis process. This holds true for the 3C analysis and in particular for the perceived autonomy during the last risk analysis. Risk assessors perceive their interaction with RM more cooperative and autonomous while risk managers perceive their interaction with risk assessors more collaborative and less autonomous.

This may be interpreted as a mirror of societal expectations. Additional empirical data collected within this project (Menning et al., forthcoming) further support this impression: when asked on their frequency of communication with risk managers, 35% of those risk assessors who reported to not communicate frequently, gave “ensuring the independence of the risk assessment” as a reason (Menning et al., forthcoming).

These differences confirm our initial assumption that current food and feed risk analysis processes within the EU face challenges. On the one hand, food and feed risk analysis processes seem to increasingly call for close iterative and flexible interaction between the actors involved. On the other hand, the organization of risk analysis processes is highly separated and coordinated. The main motivation for this strict separation was to isolate the scientific process from distorting impacts from the corporate and political realm (Calow 2014; Korinek and Veit 2015; Thompson and Bloom 2000). A greater focus on close iterative and flexible dialogue between risk assessors and risk managers can and indeed seems to be seen as a potential threat to this distinction and the scientific process in particular (Abels and Kobusch 2015; Calow 2014; Thompson and Bloom 2000).

As Abels and Kobusch (2015, 40) summarize: “scientification is crucial for the legitimacy and accountability of non‐elected experts in policy making, politicisation puts credibility of scientific expertise at risk.” Considering that the regulation of risk has “become a universal enterprise” (Löfstedt et al. 2001) and the wider dominance of the normalized risk analysis approach within that enterprise, the relevance of such barriers beyond the European food and feed context seems likely. Nonetheless, risk issues like environmental hazards, pharmaceuticals, or cybersecurity may exhibit different RA–RM dynamics. Moreover, regulatory cultures outside the EU (e.g., in Asia, Latin America, or the United States) feature different institutional structures. We encourage replications in those contexts before assuming full generalizability.

On a methodological level, our study's primary strength is the operationalization of McNamara's 2012 3C framework for food/feed risk analysis—providing one of the first structured, practitioner‐oriented instruments to quantify RA‐RM interaction.

The following limitations apply to our study: First, the perceived quality of risk analysis was entirely self‐assessed by the participants. While our results show that open and frequent communication positively impacts this perceived quality, actors engaged in highly collaborative processes may subjectively rate the efficiency and quality of their own work very highly. However, this internal satisfaction does not necessarily equate to an objectively better or unbiased system. From an external perspective—particularly concerning public trust—a system with very strong RA–RM collaboration might be viewed with skepticism if it is perceived to blur the lines of scientific independence. Future research should investigate how different degrees of internal RA–RM interaction impact the external trustworthiness and objective scientific rigor of the final RM decisions. In addition, we acknowledge a distinct structural boundary regarding the scope of our data: our empirical mapping is strictly limited to the public administration interface between regulatory assessors and regulatory managers. It does not capture the perspectives or real‐world operational constraints of industry actors, manufacturers, and retailers who are ultimately responsible for executing these food safety controls on the ground. While this broader industry network is vital to the holistic success of any regulatory framework—and its omission means our findings cannot be extrapolated to general public–private risk governance—fully evaluating industry–regulator interaction dynamics was outside the explicit scope and focus of this paper. Furthermore, future longitudinal studies could move beyond subjective self‐assessments by introducing objective, behavioral performance metrics. Specifically, researchers could explore tracking the 3C interaction profiles against the stability of policy outcomes, measuring the number of technical or legal modifications and amendments required within the first 3 years after a new regulation is implemented. A high frequency of post‐implementation corrections could indicate that the initial RA–RM interface lacked the necessary coordination or collaborative scoping required to anticipate operational bottlenecks, serving as a robust indicator of structural regulatory quality.

Second, since our 3C measures are formative and rely on self‐report; we cannot compute traditional reliability indices (Cronbach's *α*) for them. Second, with *n* = 86 included in the regression matrix we had limited power for detecting small to moderate effects in the multinomial logistic model. Third, our choice to handle the small amount of missing data via mean imputation introduces certain statistical limitations. As is well established, substituting missing values with the mean reduces overall variance, artificially inflates the degrees of freedom (DF), and can consequently overstate statistical significance. While this approach is inherently somewhat arbitrary, it was pragmatically chosen because our sample size (*n* = 95) was already working near the lower bounds of adequate power for a multinomial model containing 20 predictors. List‐wise deletion would have further degraded this power by discarding entire participant profiles over a single missing entry. Given that mean imputation was exceptionally rare—affecting a mere 5 entries out of 860 total data points (0.58%)—the structural risk of distorting our significance testing remains negligible.

Finally, because affiliation data were anonymized, we cannot assess country‐level clustering effects. To summarize, our study reveals first and foremost that RA and RM have different perceptions regarding their mutual interaction within the risk analysis process. Subsequently we were able to show that open and frequent communication positively effects the risk analysis performance. However, it is worth instantiating a differentiated view on the topic since uniform calls for more collaboration seem not to be effective for our sample.

## 6 Conclusion

Considering prominent calls to improve interaction between professional risk analysis actors, especially in the field of food and feed, we set out to map current interaction between food and feed risk assessors and managers. Mobilizing public administration literature, in particular McNamara (2012), on cooperation, coordination, and collaboration, we showed that current interaction aligns strongest with cooperation, particularly concerning separated resources, decision‐making processes, information systems and conflict resolutions. In terms of organizational design, however, respondents most dominantly described a coordinative setting. Most respondents stated that it was crucial that all partners trusted each other, which can be with collaboration.

When looking at how different forms of interaction affect the perceived risk analysis quality, we found positive effects of open and frequent communication. While this supports calls for closer communication, we did not find evidence for an overall greater performance of collaborative forms of interaction. This supports more nuanced views on the topic and consideration of the context. Analyzing such potential context dependency, we showed that especially emerging and rapid risks might call for more collaboration. However, organizations should consider wider social contexts including different perceptions across systems of science and politics and broader power dynamics. In that regard we illustrated that expectations based on normalized interpretations of the risk analysis model, in particular concerning the prescribed separation of the scientific process, can emerge as barriers for closer interaction.

While more research is needed, our article offers risk analysis actors first opportunities to explore different forms of interactions across their various dimensions, their nuanced effects on risk analysis performance and the potential need to couple appropriate interaction forms with context and desired goals. Based on our results, we suggest working towards a more individualized and fit‐for‐purpose interaction. Because our cluster analysis demonstrates that distinct interaction typologies—whether structurally coordinated, cooperative, or highly collaborative—can all result in high‐quality outcomes, organizations should not strive for a universal standard of collaboration. Accordingly, we recommend that public agencies establish required mutual information exchange or light cooperation as the primary baseline institutional default, which structurally safeguards the necessary functional separation between RA and RM. Rather than defaulting to resource‐heavy integration, RA and RM cohorts should only escalate their interaction style toward coordination or intensive collaboration when a specific dossier explicitly demands it (e.g., highly volatile crisis situations or unprecedented systemic risks), supporting this deviation with clear, well‐documented contextual arguments during the early scoping phase.

## Funding

This research was funded by the European Food Safety Authority (EFSA) under Grant Agreement No. GP/EFSA/ENCO/2020/03 (ENCOMRAN). The present publication was produced in the context of this grant. The positions and opinions presented in this article are solely those of the authors and do not necessarily represent the official views or position of EFSA.

## Data Availability

The underlying questionnaire, raw data, and data report that support the findings of this study are openly available in Zenodo at https://doi.org/10.5281/zenodo.10125145

## 1

**TABLE A1:** Regression coefficient model 3C dimensions on quality (reference for quality was the “high” group and for the 3C dimensions were the “collaboration” groups).

Variable	*Β*	Standard error	Wald	*p*	*β* (exp)
Quality (low group)					
Intercept	−16.872	2307.711	0.000	0.994	
Organizational design (cooperation)	3.535	2.076	2.900	0.089	34.295
Organizational design (coordination)	1.718	1.889	0.827	0.363	5.571
Formality of agreement (cooperation)	−0.363	1.531	0.056	0.813	0.696
Formality of agreement (coordination)	−0.377	1.881	0.040	0.841	0.686
Organizational autonomy (cooperation)	−0.878	1.824	0.232	0.630	0.415
Organizational autonomy (coordination)	−0.640	2.064	0.096	0.757	0.528
Key personnel (cooperation)	−1.476	1.501	0.966	0.326	0.229
Key personnel (coordination)	−2.213	1.768	1.566	0.211	0.109
Information sharing (cooperation)	1.145	1.398	0.670	0.413	3.142
Information sharing (coordination)	5.961	2.112	7.693	0.005	387.921
Decision making (cooperation)	−2.171	1.478	2.155	0.142	0.114
Decision making (coordination)	0.004	2.459	0.000	0.999	1.004
Turf issues (cooperation)	−0.007	1.606	0.000	0.997	0.993
Turf issues (coordination)	52.204	8530.642	0.000	0.995	0.001
Resource allocation (cooperation)	−19.556	1650.821	0.000	0.991	0.003
Resource allocation (coordination)	−19.256	1650.822	0.000	0.991	0.004
Trust (cooperation)	−0.537	1.398	0.147	0.701	0.585
Trust (coordination)	1.398	1.538	0.827	0.363	4.048
Systems thinking (cooperation)	35.144	2837.380	0.000	0.990	0.001
Systems thinking (coordination)	−0.704	3699.248	0.000	1.000	0.495
Quality (moderate group)					
Intercept	17.865	1650.821	0.000	0.991	
Organizational design (cooperation)	1.165	1.012	1.324	0.250	3.206
Organizational design (coordination)	1.118	0.926	1.455	0.228	3.057
Formality of agreement (cooperation)	0.179	0.806	0.050	0.824	1.196
Formality of agreement (coordination)	−0.357	1.130	0.100	0.752	0.699
Organizational autonomy (cooperation)	−0.736	1.145	0.413	0.521	0.479
Organizational autonomy (coordination)	−1.813	1.363	1.769	0.184	0.163
Key personnel (cooperation)	−0.999	0.847	1.392	0.238	0.368
Key personnel (coordination)	0.660	1.002	0.433	0.510	1.935
Information sharing (cooperation)	−0.566	0.812	0.486	0.486	0.568
Information sharing (coordination)	−0.053	1.525	0.001	0.972	0.948
Decision making (cooperation)	−1.026	1.110	0.854	0.355	0.359
Decision making (coordination)	−36.077	4089.006	0.000	0.993	0.002
Turf issues (cooperation)	−0.841	1.310	0.419	0.518	0.431
Turf issues (coordination)	−0.595	0.000	0.000	1.000	0.551
Resource allocation (cooperation)	−15.210	1650.820	0.000	0.993	0.002
Resource allocation (coordination)	−16.949	1650.822	0.000	0.992	0.004
Trust (cooperation)	0.422	0.966	0.191	0.662	1.525
Trust (coordination)	1.740	0.982	3.144	0.076	5.699
Systems thinking (cooperation)	−1.235	1.139	1.176	0.278	0.291
Systems thinking (coordination)	0.692	1.548	0.200	0.655	1.999

**TABLE A2:** Regression coefficient model 3C dimensions on quality (reference for quality was the “high” group and for the 3C dimensions were the “collaboration” groups).

Two‐item dimension	*r*	*α*_2_
Formality of agreement	−0.25	−0.45
Turf issues	−0.78	−7.05
Resource allocation	0.32	0.49
